# Reciprocal signals between microglia and neurons regulate α-synuclein secretion by exophagy through a neuronal cJUN-N-terminal kinase-signaling axis

**DOI:** 10.1186/s12974-016-0519-5

**Published:** 2016-03-08

**Authors:** Dan Ploug Christensen, Patrick Ejlerskov, Izabela Rasmussen, Frederik Vilhardt

**Affiliations:** Department of Cellular and Molecular Medicine, Faculty of Health Sciences, University of Copenhagen, 3C Blegdamsvej, 2200 Copenhagen N, Denmark; Biotech Research and Innovation Centre, University of Copenhagen, Ole Maaløes Vej 5, 2200 Copenhagen N, Denmark

**Keywords:** Inflammation, Synucleinopathy, Microglia, Neuronal secretion, JNK, Parkinson’s disease

## Abstract

**Background:**

Secretion of proteopathic α-synuclein (α-SNC) species from neurons is a suspected driving force in the propagation of Parkinson’s disease (PD). We have previously implicated exophagy, the exocytosis of autophagosomes, as a dominant mechanism of α-SNC secretion in differentiated PC12 or SH-SY5Y nerve cells. Here we have examined the regulation of exophagy associated with different forms of nerve cell stress relevant to PD.

**Results:**

We identify cJUN-N-terminal kinase (JNK) activity as pivotal in the secretory fate of autophagosomes containing α-SNC. Pharmacological inhibition or genetic (shRNA) knockdown of JNK2 or JNK3 decreases α-SNC secretion in differentiated PC12 and SH-SY5Y cells, respectively. Conversely, expression of constitutively active mitogen-activated protein kinase kinase 7 (MKK7)-JNK2 and -JNK3 constructs augment secretion. The transcriptional activity of cJUN was not required for the observed effects. We establish a causal relationship between increased α-SNC release by exophagy and JNK activation subsequent to lysosomal fusion deficiency (overexpression of Lewy body-localized protein p25α or bafilomycin A1). JNK activation following neuronal ER or oxidative stress was not correlated with exophagy, but of note, we demonstrate that reciprocal signaling between microglia and neurons modulates α-SNC secretion. NADPH oxidase activity of microglia cell lines was upregulated by direct co-culture with α-SNC-expressing PC12 neurons or by passive transfer of nerve cell-conditioned medium. Conversely, inflammatory factors secreted from activated microglia increased JNK activation and α-SNC secretion several-fold in PC12 cells. While we do not identify these factors, we extend our observations by showing that exposure of neurons in monoculture to TNFα, a classical pro-inflammatory mediator of activated microglia, is sufficient to increase α-SNC secretion in a mechanism dependent on JNK2 or JNK3. In continuation hereof, we show that also IFNβ and TGFβ increase the release of α-SNC from PC12 neurons.

**Conclusions:**

We implicate stress kinases of the JNK family in the regulation of exophagy and release of α-SNC following endogenous or exogenous stimulation. In a wider scope, our results imply that microglia not only inflict bystander damage to neurons in late phases of inflammatory brain disease but may also be active mediators of disease propagation.

## Background

Within recent years, it has been convincingly shown that propagating protein malconformation disease in the mouse brain can be achieved by inoculation of recombinant α-synuclein (α-SNC) or amyloid-β protein aggregates [1]. Propagation ensued along anatomically connected fiber tracts and may reflect the propensity of Parkinson’s disease (PD) to progress along neuronal trajectories in the human brain as delineated by Braak et al. [2]. These phenomenological studies are supported by a growing body of in vitro and in vivo experiments demonstrating interneuronal transmission of endogenously produced and secreted α-SNC [1–7]. Once taken up, the internalized aggregates engage in proteopathic templating for the perpetuation of malconformation disease [8]. Thus, there is great interest in understanding the mechanisms that govern the release of α-SNC monomers and aggregates under physiological and pathological conditions, because therapeutic modulation of extracellular α-SNC could be a way to prevent the propagation of PD once diagnosed. Several groups have documented neuronal α-SNC secretion [3, 9–14], but knowledge of the mechanisms that govern secretion is limited. Both exocytosis of autophagosomes/amphisomes [10] or late endosomes [11] containing α-SNC have been proposed as vehicles of α-SNC secretion, but the regulatory mechanisms that drives exocytosis, rather than the classical trafficking pattern to lysosomal fusion and degradation, are largely unknown.

Nerve cells depend on autophagy for proteostasis and survival due to their post mitotic nature [15]. In PD and other malconformation brain diseases, where pathology is associated with abnormal protein folding and aggregate accumulation, autophagy is activated [16, 17] to compensate for the often compromised proteasomal function. While α-SNC is taken up by both macroautophagy and chaperone-mediated autophagy, modified or aggregated forms of α-SNC have also been found to partially inhibit the very same pathways [16–21]. There is also mounting evidence that lysosomal function is compromised in PD and other brain diseases [22]. Disturbed trafficking of hydrolases and other lysosomal proteins, including the vacuolar proton ATPase essential for acidification, results in a reduced complement of lysosomes and their enzymatic activities [23–27]. In addition, the sequestration of fusion machinery or the aberrant sorting of PD-associated sorting receptors have been implicated [25, 27, 28] altogether compromising the ability for an uninterrupted flow through the autophagosomal and endosomal pathways.

We have recently described how the small, disordered protein tubulin polymerization-promoting protein (p25α) perturbs the autophagosomal pathway in parkinsonergic nerve cell models. This small protein, normally expressed in oligodendrocytes, is ectopically expressed in dopaminergic neurons in PD, is co-localized with α-SNC in Lewy bodies, and has a strong propensity to aggregate α-SNC [29]. Therefore, p25α upregulates autophagy and increases the autophagosomal uptake of α-SNC [10]. However, through its inhibition of histone deacetylase 6 [30], required for actin remodeling [31], p25α also partially blocks fusion of autophagosomes and amphisomes, the fusion organelle of an autophagosome and a late endosome, with lysosomes [10]. In turn, this greatly increases the secretion of α-SNC due to the exocytosis of amphisomes and/or autophagosomes. Accumulation of autophagosomes and amphisomes is cytotoxic [32], and therefore secretion of α-SNC via exophagy can be regarded as a last resort to maintain proteostasis and cell viability [10].

To study autophagy and exophagy of α-SNC in vitro, we have used the commonly known nerve cell lines PC12 and SH-SY5Y cells often used as cellular models of dopaminergic neurons. PC12 cells derive from a rat pheochromocytoma and can be differentiated to chatecholaminergic neuron-like cells in low serum and addition of nerve growth factor (NGF), while human neuroblastoma cell line SH-SY5Y can be differentiated to chatecholaminergic, but predominantly noradrenergic, nerve cells under low serum concentrations in the presence of either all-trans-retinoic acid (ATRA) or brain-derived neurotrophic factor (BDNF). We have transduced these cell lines to conditionally express α-SNC (wt or A30P) with or without concurrent p25α expression. During our travail, we observed that p25α expression in both cell types causes the persistent activation of cJUN-N-terminal kinase (JNK) in the absence of overt cell death [10]. The JNK family consists of a number of isoforms divided into JNK1, JNK2, and JNK3 subtypes. JNK1 and JNK2 are expressed ubiquitously, but JNK3 is primarily expressed in the brain [33]. All three individual JNK knockouts in mice are viable. While JNK1 is involved with neuronal housekeeping functions such a dendritic arborization, JNK2 and JNK3 are activated primarily in response to stressful conditions [33]. Often protracted JNK activation will result in apoptosis but the anti- and pro-apoptotic functions of JNK isoforms are controlled by the mitogen-activated protein kinase kinase kinase dual leucine zipper kinase (DLK) [34] and the subcellular distribution (nucleus versus cytosol) of activated JNK [33, 35].

JNK is activated in response to inflammatory signaling. The transmission hypothesis of malconformation disease propagation does however not address the role of microglia, the resident central nervous system (CNS) immune cells, which are essential for development and progression of neurodegenerative diseases [36]. In the mature brain, microglia exist in a surveying state [37] but any deviation from CNS homeostasis, immunological stimuli, or signaling from neurons can activate microglia [36, 37]. However, protracted activation of microglia, basically due to their inability to eradicate the initiating stimulus, causes neurotoxic effects by excess production of cytotoxic factors such as superoxide [38] and tumor necrosis factor α (TNFα) [39]. Notably, aggregated α-SNC has recently been shown to activate microglia through Toll-like receptor 2 (TLR2) [40] and complement receptor 3 (CR3) [41] signaling.

In the present work, we demonstrate that activation of neuronal stress kinases JNK2 and/or JNK3 is essential for the exophagosomal release of α-SNC from differentiated PC12 and SH-SY5Y nerve cells. We find that JNK is activated not only as a consequence of endogenous stress relating to lysosomal fusion deficiency but also following inflammatory signaling from co-cultured microglia, which were themselves activated by the α-SNC secreting neurons. In both instances, activated JNK supports an augmented secretion of α-SNC from neurons. In a broader scope, our results suggest that inflammatory microglia also in vivo could modulate release of proteotoxic α-SNC aggregates from neurons and thereby disease propagation, in a manner mechanistically different from the well-documented bystander damage to neighboring neurons in later phases of disease.

## Methods

### Antibodies and chemical reagents

Antibodies used were mouse monoclonal anti-α-synuclein antibody (BD Transduction Laboratories, Franklin Lakes, NJ); rabbit polyclonal anti-phospho-SAPK/JNK (Thr183/Tyr185) antibody (#9251, Cell Signaling, Danvers, MA); rabbit polyclonal anti-phospho-cjun (#2592, Epitomics, San Francisco, CA); mouse monoclonal anti-β-actin (A-1978, Sigma-Aldrich, St Louis, MO); rabbit polyclonal anti-p25α antibody (Enzo Life Sciences, Farmingdale, NY); mouse monoclonal anti-FLAG tag antibody (#A00187-200, Genscript, Aachen, Germany); mouse monoclonal anti-FLAG tag antibody (clone M2, Sigma-Aldrich); mouse monoclonal anti-JNK1 antibody (F-3) (sc-1648, Santa Cruz Biotechnology, Dallas, TX); rabbit polyclonal anti-JNK2 antibody (#4672, Cell Signaling); rabbit monoclonal anti-JNK3 antibody (#2305, Cell Signaling); mouse monoclonal anti-CHOP antibody (Abcam, ab11419); rabbit polyclonal anti-Nrf2 antibody (sc-22810, Santa Cruz); rat monoclonal anti-cluster of differentiation 11b (CD11b) antibody (MCA711, AbD Serotec, Puchheim, Germany); and rabbit polyclonal anti-TLR2 antibody (sc-10739, Santa Cruz). Salubrinal, SP600125, phorbol 12-myristate 13-acetate (PMA), lipopolysaccharide serotype O:55 (LPS), doxycycline, puromycine, Triton X-100, Tween-20, NADH, sodium pyruvate, trichloroacetic acid (TCA), phosphatase and protease inhibitors, and H_2_O_2_ were all from Sigma. Human and rat tumor necrosis factor (TNF)α, rat interleukin (IL)34, rat transforming growth factor (TGF)β1, rat interferon (IFN)β1, human brain-derived neurotrophic factor (BDNF), and granulocyte macrophage-colony stimulating factor (GM-CSF) were all from Peprotech (Rocky Hill, NJ). Alexa 488-, 568-, or 633-conjugated goat anti-mouse or anti-rabbit antibodies were from Molecular Probes (Life Technologies, Grand Island, NY); horseradish peroxidase (HRP)-conjugated swine or goat anti-mouse or rabbit secondary antibodies were from Dako (Glostrup, Denmark); ToPro-3 iodide for nucleus staining was from Molecular Probes.

### Cell culture and neuronal differentiation

The rat pheochromocytoma cell line PC12 (ATCC) was seeded on collagen-coated culture dishes and cultured in DMEM containing 10 % horse serum, 5 % fetal calf serum, and 1 % penicillin and streptomycin at 37 °C in 5 % CO_2_. For experiments, PC12 cells were seeded at a density of 45,000 cells/cm^2^ and differentiated in DMEM containing 2 % horse serum, 1 % penicillin and streptomycin, and 100 ng/ml nerve growth factor (NGF) 2.5S subunit, (Serotec, Raleigh, NC) for 2 days before transgene expression was induced for an additional 2 days. Human neuroblastoma SH-SY5Y cells were cultured in DMEM containing 10 % fetal calf serum and 1 % penicillin and streptomycin at 37 °C in 5 % CO_2_. For experiments, SH-SY5Y cells were serum starved at high cell density for 5 days and then reseeded and differentiated with 10 μM all-trans-retinoic acid (ATRA) or 10 μg/ml BDNF in serum-free medium for 6–8 days to obtain a neuron-like phenotype. Transgene expression was induced in the last 48 h before experimentation. PC12 cells conditionally (tet-on) expressing human α-SNC w/wo p25α have previously been established and described, as have SH-SY5Y cells stably expressing α-SNC A30P w/wo conditional expression of p25α [10]. Purification and culture of primary microglia from neonatal rats was performed as previously described [42]. The murine microglial cell line Ra2 (licensed by the Japan Science and Technology Agency, Patent ID US6.673,6,5; JP3410738; EP10/602,234) was kindly provided by Dr. Makoto Sawada (Dept. of Brain Function, Nagoya University, Nagoya, Japan) and maintained in MEM with 10 % FCS, 1 ng/ml GM-CSF, and 5 μg/ml bovine insulin [10]. Ra2-gp91^phox^ cells with gp91phox under the control of an elongation factor (EF) promotor have previously been described [42]. In all instances, doxycycline (100-200 ng/ml) was used to induce transgene expression for a minimum of 48 h.

### Lentivirus production and transduction

Lentivectors SIN-W-PGK-ASK1-K709R and SIN-W-PGK-Flag-Δ169c-Jun [43] were a generous gift of Dr. Deglon (CEA, Institute of Biomedical Imaging and Molecular Imaging Research Center, Orsay, France). Lentiviral vectors pLKO.1 JNK1 shRNA (TRCN0000055115), pLKO.1 JNK2 shRNA (TRCN0000012590), and pLKO.1 JNK3 shRNA (TRCN0000012634) were all from Open Biosystems (Dharmacon, Lafayette, CO). Control vector pLKO.1 scrambled (Scr) shRNA was from Sigma (#SHC002). Lentiviral particles for infection were produced as previously described [44] before superinfection of PC12 cells conditionally expressing α-SNC alone (PC12 α-SNC) or with p25α (PC12 α-SNC/p25α) and SH-SY5Y cells expressing α-SNC_A30P_ alone (SH-SY5Y α-SNC_A30P_) or with conditional p25α expression (SH-SY5Y α-SNC_A30P_/p25α). Cells stably expressing scrambled shRNAs were selected with 0.2 μg/ml puromycin for at least 5–7 days before beginning differentiation and experimentation. To generate Ra2 microglia constitutively expressing the encoded H_2_O_2_ sensor HYPER3 [45] (generously provided by Dr. Belousov, Shemyakin-Ovchinnikov Institute of Bioorganic Chemistry, Moscow, Russia), complementary DNA (cDNA) was PCR-cloned into the BamHI/XhoI-restricted lentiviral vector pHR-cPPT.CMV.W expressing cDNA under the control of the constitutive cytomegalovirus (CMV) promotor.

### Transient transfections with constitutive active JNK isotypes

Plasmids expressing Flag-tagged constitutively active versions of JNK1-3 (pCDNA3 Flag MKK7B2Jnk1a1, pCDNA3 Flag MKK7B2Jnk2a2 and pCDNA3 Flag MKK7B2Jnk3a1) and kinase dead mutant JNK1 (pCDNA3 Flag MKK7B2JNK1a1(AFP)) are described in [46] and were obtained through Addgene (Cambridge, MA). Transient transfection was performed with differentiated SH-SY5Y α-SNC_A30P_ cells utilizing the Lipofectamine 2000 (Life Technologies) reagent according to the manufacturer’s instructions. Conditioned medium was collected, and cells were lysed for analysis by western blotting for p-JNK, Flag, and α-SNC, respectively, after an overnight transfection period.

### Western blotting (WB)

Cells were lysed in lysis buffer (100 mm NaCl, 50 mm Tris-HCl, 1 mm EGTA, 10 mmMgCl_2_, pH 7.2) containing 1 % Triton X-100, phosphatase, and protease inhibitor mixture for 5 min at room temperature and thereafter kept on ice. Cell lysates were centrifuged at 16,100×*g* for 5 min at 4 °C, and protein concentrations of the supernatant were determined with D_c_ protein assay (Bio-Rad, Copenhagen, Denmark), before the addition of Laemmli buffer and loading of equivalent protein quantities on SDS-polyacrylamide gels. Following transfer to PVDF membranes, western blotting was performed using chemiluminescent HRP detection substrate (Millipore, Hellerup, Denmark). Specifically, for p-JNK in differentiated PC12 cells exposed to Ra2-conditioned medium (Fig. 6e, f), Ra2 cells were changed to HBSS ± LPS (0.5 μg/ml) ± NGF for 6 h before conditioned HBSS was collected from Ra2 monoculture and centrifuged 6000 rpm at 4 °C for 3 min prior to transfer to differentiated PC12 cell monoculture for a 6-h incubation. After 6 h, PC12 conditioned medium was recovered and cells lysed and prepared for western blot as described. All western blot bands were quantified with ImageJ or Image Lab.

### Trichloroacetic acid protein precipitation

Conditioned medium was harvested and centrifuged at 800×*g* for 5 min, 4 °C, before 20 % (v/v) trichloroacetic acid (TCA) was added to the supernatant and incubated on ice for 10 min. The protein precipitates were pelleted by centrifugation (16,100×*g*, 10 min, 4 °C) and washed four to five times in ice-cold acetone until the pellet appeared clear white. The pellets were dried at 95 °C for 20 min, dissolved in ×2.5 Laemmli buffer, boiled for 20 min at 95 °C, and subsequently processed for western blotting.

### Microglia oxidant production

Differentiated PC12 neurons contained in six-wells (ca. 60 % confluency) were incubated with 750.000/well Ra2 microglia expressing HYPER3 in PC12 medium MEM with additives (as described above). Cells were co-cultured overnight without (control) or with 100 ng/ml LPS. The next day, cells were flushed off the culture vessel with HBSS and immediately analyzed by flow cytometry on a Beckton Dickinson FACSaria using the 488 nm laser for excitation of HYPER3. To take advantage of ratiometric HYPER3 measurements, we also performed a microtiter-based fluorescence assay. In this format, conditioned medium from PC12 cells was applied to adherent Ra2 microglia expressing HYPER3 contained in ELISA wells (50,000/well) for 2 hours before the analysis of HYPER3 fluorescence using a FLEX station at 37 °C with excitation at 485/420 nm and emission at 516 with cutoff filter set at 495 nm. HYPER3 measurements were performed with Ra2 microglia receiving only PC12 conditioned medium (basal) or in addition also 100 ng/ml PMA. Exogenous H_2_O_2_ (100 μM) and DTT (100 mM) were used to obtain maximal oxidation or reduction of the HYPER3 probe for comparison.

### Lactate dehydrogenase (LDH) assay

To evaluate cell death during our experiments, we collected a small fraction of the aspirated media to be used for LDH assay. Samples were stored at 4 °C until assay was performed and maximally 4–6 days. Aspirated media were centrifuged 6000 rpm, 5 min, at 4 °C to remove cell debris before applying 25-μl sample in duplicate to a 96-well plate. Two hundred microliters freshly prepared reagent solution (0.08 M Trizma Base, 0.2 M NaCl, 148.5 μg/ml NADH, and 93.5 μg/ml sodium pyruvate, ph 7.2) was added, and immediately, Abs340nm was measured for approximately 20 min at room temperature (1 reading each 20–30 s). The slope of the resulting Abs340nm change over time was calculated and normalized to protein amounts for each sample. All measurements were related to total cellular content of LDH obtained by the addition of 0.1 % Triton X-100 to control wells to release all LDH to medium.

### Immunofluorescence/confocal microscopy

PC12/Ra2 co-cultures were washed once in Hanks’ balanced saline solution and fixed in a phosphate buffer containing 2 % paraformaldehyde, pH 7.4, for 30 min. Immunofluorescence was essentially performed as previously described [44], and images were acquired with a Zeiss LSM510 confocal laser scanning microscope with a C-Apochromat ×63, 1.4 NA oil immersion objective, using the argon 488-nm and the helium/neon 543- and 633-nm laser lines for excitation of Alexa 488, 568, and 633, respectively. Confocal sections of 0.8–1.0 μm were collected and saved as 512 × 512-pixel or 1024 × 1024-pixel images at 12-bit resolution. The same microscopy settings were used for obtaining all images relating to the same series of experiments, and images were prepared and compiled without digital manipulation.

### Statistical analyses

Comparison between two groups was done by Student’s *t* test. Comparisons of more than two groups were done by one- or two-way ANOVA with either Tukey’s (comparing every mean with every other mean) or Dunnett’s correction (comparing every mean with a control mean) for multiple comparisons. A *p* value <0.05 was considered statistically significant. All data are graphically represented as means + SEM or given as means ± SD. For Western blotting, all calculations were performed with actin-normalized integrated optic density (IOD) where applicable or with raw IOD values. Statistical evaluation was performed with Graphpad Prism.

## Results

### JNK regulates neuronal secretion of α-SNC

**Fig. 1:**
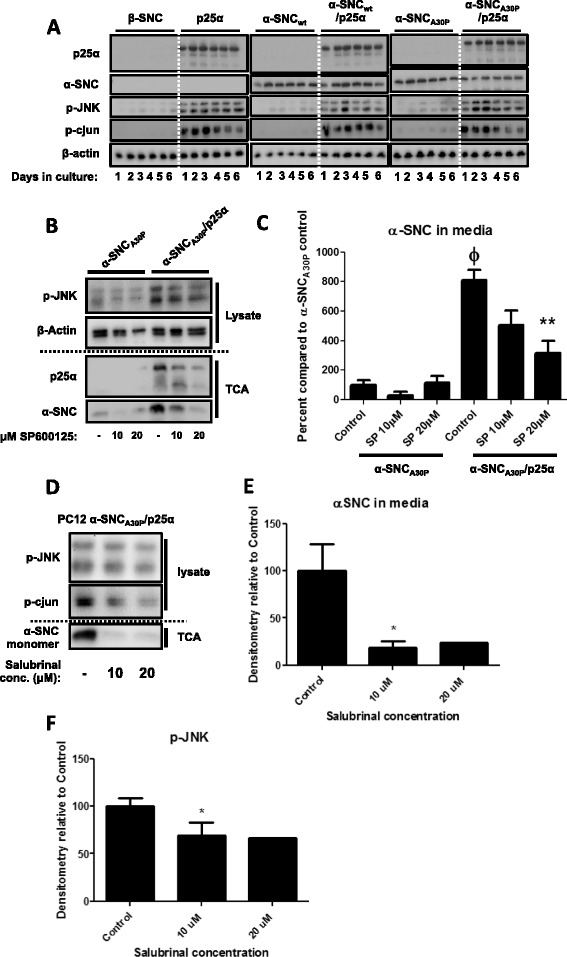
Pharmacological JNK inhibition reduces α-SNC secretion from neurons. **a** Lysates of differentiated PC12 cells expressing α-SNC and/or p25α for 1–6 days after transgene induction were western blotted with antibodies as indicated. The blots shown are representative of three independent experiments. **b** PC12 cells expressing α-SNC_A30P_ or α-SNC_A30P_/p25α were treated with JNK inhibitor SP600125 (10 and 20 μM) for 48 h before subjecting whole cell lysates and protein precipitated from conditioned media (TCA) to western blotting as indicated. **c** Quantification of **b**. Data are presented as mean + SEM (*N* = 3) normalized to “α-SNC_A30P_ Control” (ϕ: *p* < 0.05 compared to “α-SNC_A30P_ Control”; **p < 0.01 compared to “α-SNC_A30P_/p25α Control”). **d** PC12 cells expressing α-SNC_A30P_/p25α were treated with salubrinal (10 and 20 μM) for 48 h before whole cell lysate, and protein precipitated from conditioned medium (TCA) were subjected to WB analysis. **e**–**f** Quantifications of **d**. Data are presented as mean + SEM (*N* = 3) percent normalized to untreated control cells (*p < 0.05 compared to “Control”)

We previously noted that p25α expression in differentiated PC12 nerve cells caused massive and protracted activation of JNK and its downstream target cJUN alongside a greatly increased emission of α-syn to the surroundings. We therefore analyzed JNK activation in more detail. To detect activated JNK, we performed western blotting of phosphorylated (p)-JNK and its downstream target cJUN in whole cell lysates from differentiated PC12 cells expressing various combinations of α-SNC (wt or A30P) and p25α over a 6-day culture period after transgene induction with doxycycline (Fig. 1a). When expressing α-SNC_wt_ or α-SNC_A30P_ alone, there was a small increase in levels of p-JNK and p-cJUN compared to control cells transduced with β-synuclein (β-SNC). In contrast, p25α caused a dramatic and persistent activation of JNK and downstream target cJUN regardless of co-expression of α-SNC.

JNK is known to be involved in regulated trafficking of vesicular elements along microtubuli tracks [33]. To investigate if activated JNK plays a role in secretion of α-SNC, we first tested the effect of SP600125, which is a potent and reversible inhibitor of JNK. Differentiated PC12 cells expressing α-SNC_A30P_ either alone or with p25α were incubated with 10 or 20 μM SP600125 for 48 h (Fig. 1b), and the conditioned medium was analyzed for secreted α-SNC by TCA precipitation and western blotting. Following exposure to 10 or 20 μM SP600125, p-JNK was decreased without affecting cell death (measured by caspase 3 activation, data not shown) in a dose-dependent manner in cells expressing α-SNC_A30P_/p25α, while the response was biphasic in PC12 α-SNC_A30P_ cells. ER stress and induction of the unfolded protein response (UPR) is a common feature of protein malconformation disease. As JNK can be activated following ER stress [47], we therefore also examined the effect of salubrinal, which inhibits dephosphorylation of phosphorylated eukaryotic Initiation Factor 2α (eIF2α), and thereby prolongs the UPR to relieve in part the cytotoxicity of ER stress [48]. As shown in Fig. 1d–f, 10–20 μM salubrinal decreased levels of p-JNK as well as secretion of α-SNC from PC12 α-SNC/p25α expressing cells.

### JNK activation is not due to ER stress in p25α-expressing cells

**Fig. 2:**
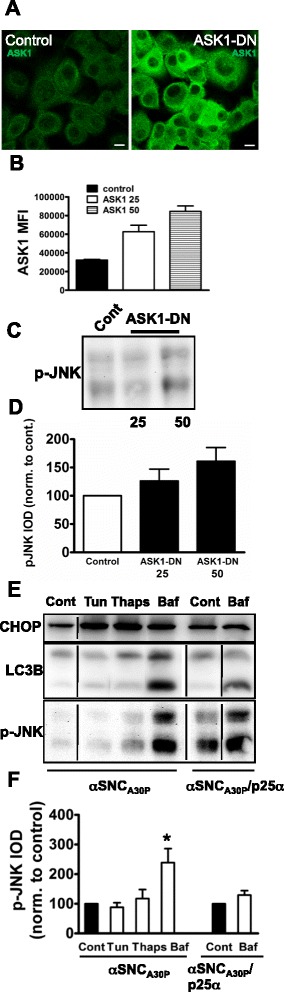
ER stress does not mediate JNK activation in differentiated PC12 or SH-SY5Y nerve cells. **a**–**d** PC12 cells expressing α-SNC/p25α were transduced with control vector or dominant-negative ASK1-K709R (ASK1-DN) with a viral dose of 25 or 50 μl. **a** Two days post-infection cells transduced with control or 50 μl ASK1-DN vector were prepared for immunofluorescence with anti-ASK1 antibodies. *Bar*, 10 μm. **b** PC12 α-SNC/p25α cells transduced with control or ASK1-DN vector were stained with anti-ASK1 antibodies and analyzed by flow cytometry. Results are expressed as mean fluorescent intensity (MFI) of ASK1 staining (mean and SEM, *N* = 2). **c** Cell lysates prepared from transduced PC12 cells were western blotted using anti-p-JNK antibodies. **d** Densitometric quantitation of p-JNK western bands from above. Results are expressed as mean integrated optical density (IOD) normalized to control-transduced cells (mean and SEM, *N* = 4). **e**, **f** ATRA-differentiated SHSY-5Y cells expressing α-SNC_A30P_ alone or together with p25α were treated overnight with tunicamycin (Tun; 700 nM), thapsigargin (Thaps; 25 nM), or bafilomycin A (Baf; 20 nM). **e** Cell lysates were then western blotted with anti-CHOP, anti-LC3B, and anti-p-JNK antibodies. All lanes shown for each respective antibody were derived from the same membrane. **f** Densitometric quantitation of p-JNK western bands from above. Results are expressed as mean and SEM integrated optical density (IOD) normalized to (respective) control-transduced cells (*N* = 3, *p < 0.05 by Student *t* test compared to control)

Our observations that salubrinal decreased p-JNK levels and α-SNC release prompted us to investigate whether induction of ER stress is sufficient to activate JNK in p25α-expressing nerve cells. ER stress mediates JNK activation through an inositol requiring ezyme-1α (IRE1α)—TNFα receptor-associated factor 2 (TRAF2)—apotosis signal-regulating kinase-1 (ASK1) signaling axis [49]. Incidentally, ASK1 is also a major activator of JNK signaling in response to oxidative stress [50]. We therefore first examined the effect of expression of dominant-negative ASK1-K709R in differentiated PC12 nerve cells. As shown in Fig. 2a, b, ASK1-K709R was efficiently transduced into PC12 cells; however, p-JNK levels were in fact moderately upregulated in these cells (Fig. 2c, d), excluding the IRE1α-TRAF2-ASK1 axis in the activation of JNK. Secondly, we exposed ATRA-differentiated SH-SY5Y cells expressing α-SNC_A30P_ to the ER stressors tunicamycin and thapsigargin, which induced the UPR as evidenced by upregulation of C/EBP homologous protein (CHOP) (Fig. 2e). However, neither agent induced a significant change in the levels of p-JNK (Fig. 2e, f), and in fact, in BDNF-differentiated SH-SY5Y cells, these agents moderately decreased JNK activity (see Fig. 9b, e). In contrast, we note that incubation with bafilomycin A, which inhibits fusion of lysosomes with late endosomal or autophagosomal organelles, caused accumulation of the autophagosomal marker LC3B-II (Fig. 2e) and increased p-JNK levels more than twofold in α-SNC_A30P_-expressing SH-SY5Y cells (*p* < 0.05). Bafilomycin A inhibits the vacuolar proton ATPase, and as a consequence, inhibits late endosome or amphisome fusion with lysomes, thereby mimicking the effect of p25α overexpression [10]. Therefore, the effect of bafilomycin A was expectedly reduced in SH-SY5Y cells co-expressing p25α (Fig. 2f). In conclusion, the observed activation of JNK in PC12 and SHSY-5Y nerve cells is not due to ER stress or oxidative stress but rather related to perturbation of membrane trafficking in the endosomal and/or autophagosomal pathways.

### shRNA knockdown of JNK2 or JNK3 reduces neuronal α-SNC secretion

**Fig. 3:**
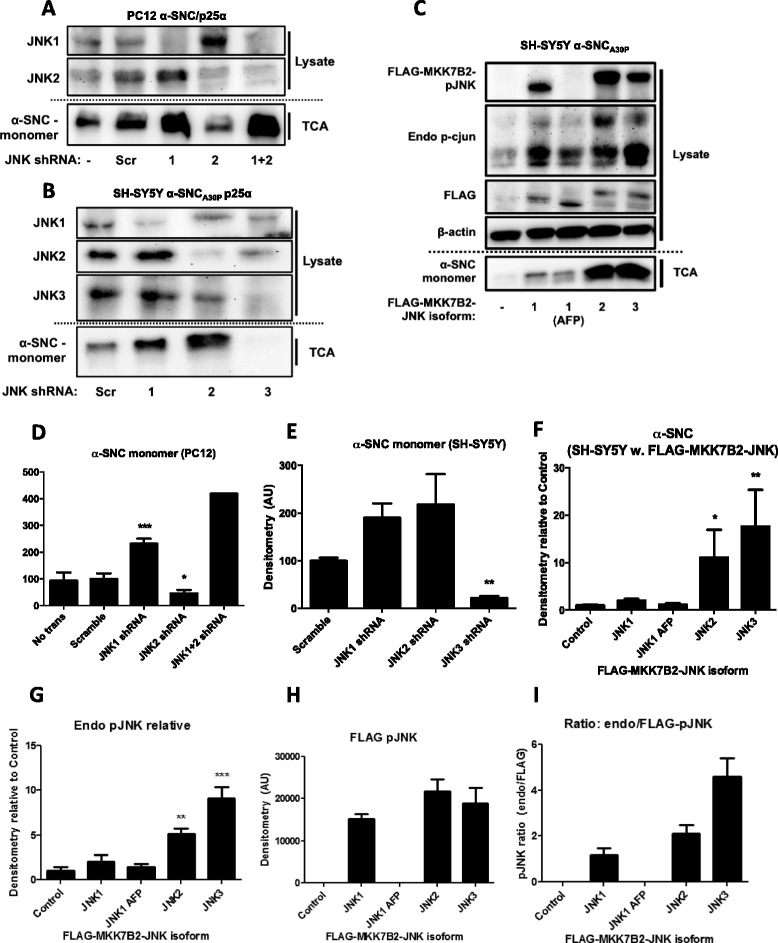
JNK2 or JNK3 shRNA reduce while JNK activation increases α-SNC secretion from neurons. **a** PC12 cells expressing α-SNC/p25α and JNK1, JNK2, or JNK1+2 shRNA were lysed after 48 h transgene induction and whole cell lysates and conditioned media (TCA) were subjected to western blotting against JNK1, JNK2, and α-SNC. Showing representative images from two to three independent experiments (we performed only two experiments with JNK1+2 shRNA expressing cells). **b** Differentiated SH-SY5Y cells expressing α-SNC/p25α and JNK1-3 shRNA were lysed after 48-h transgene induction, and whole cell lysates and conditioned media (TCA) were western blotted as indicated. The blots shown are representative of three independent experiments. **c** Differentiated SH-SY5Y cells expressing α-SNC/p25α were transfected with plasmids encoding constitutively active (FLAG-MKK7B2-JNK1-3) JNK isoforms or a kinase dead version of JNK1 (AFP) as control. The day after transfection, whole cell lysates and conditioned media (TCA) were western blotted as indicated. The blots shown are representative of three independent experiments. **d** Quantification of **a** α-SNC present in conditioned medium. Data are shown as mean + SEM (*N* = 3) percent relative to control transfected with scrambled (Scr) shRNA (*p < 0.05; ***p < 0.001 compared to “Scrambled”). **e** Quantification of **b** α-SNC present in conditioned medium. Data are shown as mean + SEM (*N* = 3) percent relative to control transfected cells (**: p < 0.01 compared to “Scramble”). **f**-**h** Quantification of (C) α-SNC present in conditioned medium (TCA), endogenous (Endo) p-JNK, and FLAG-MKK7B2-JNK1-3 (FLAG) p-JNK. Data are presented as mean + SEM (*N* = 3) normalized to control cells transfected with an empty vector (*p < 0.05; **p < 0.01; ***p < 0.001 compared to “Control”). **i** Calculated ratio of p-JNK of endogenous to FLAG-MKK7B2-JNK1-3 densitometric western blot signals

To identify the JNK isoform(s) in question, we established PC12 and SH-SY5Y cell lines conditionally expressing α-SNC_wt_/p25α or α-SNC_A30P_/p25α, respectively, together with stable expression of JNK1, JNK2, or JNK3 shRNA. These cells were then differentiated to mature neurons before lysate and conditioned medium were collected for analysis. We confirmed the knockdown of specific JNK isoforms by western blotting (Fig. 3a, b) and analyzed α-SNC secretion by TCA precipitation of the media and western blotting. We found that knockdown specifically of JNK2 in PC12 cells (Fig. 3a, d) and JNK3 in SH-SY5Y cells (Fig. 3b, e) significantly decreased α-SNC secretion compared to control cells expressing scrambled shRNA. Of note, PC12 cells do not express JNK3 [51]. In differentiated PC12 cells, JNK1 shRNA significantly increased α-SNC secretion, while JNK1 and JNK2 knockdown in the differentiated SH-SY5Y cells also increased secretion. To further substantiate that active JNK in neurons is associated with α-SNC secretion, we utilized dominant-positive JNK1, JNK2, and JNK3 fusion constructs (FLAG-MKK7B2-JNK1/2/3). The FLAG-tagged constructs encode JNK fused to a constitutively active upstream mitogen-activated protein kinase kinase 7 (MKK7), which persistently activates the JNK moiety [46]. The FLAG-MKK7B2-JNK1 (AFP) construct is a JNK kinase dead control. Constructs were transiently transfected into differentiated SH-SY5Y cells expressing α-SNC_A30P_ (Fig. 3c, f–i) to observe whether constitutive JNK activity could replicate the effect of p25α on α-SNC secretion. The kinase dead AFP-construct afforded no change in α-SNC release relative to control cells, active JNK1 moderately increased secretion, but notably JNK2 and JNK3 isoform transfection many-fold increased the release of α-SNC (*p* < 0.05) (Fig. 3f). Both transfected JNK (FLAG-MKK7B2-p-JNK, Fig. 3c, h) and endogenous JNK (endo p-JNK, Fig. 3c, g) phosphorylation were increased, and the highest amounts of endogenous p-JNK were found after JNK2 and JNK3 transfections (*p* < 0.05 compared to control). No significant changes in cell death were detected as assayed by lactate dehydrogenase (LDH) activity in the medium (data not shown). The ratio of endogenous p-JNK relative to FLAG-tagged-p-JNK (Fig. 3i) proved to be highest for the JNK3 fusion construct, suggesting that similar amounts of transfected JNK activity (FLAG-p-JNK) pertaining to different isoforms (JNK1-3) caused the highest amount of endogenous JNK activation (endo p-JNK) for the JNK3 isoform. Hence, JNK3 appears as the most efficient signaling entity for α-SNC secretion of the three investigated.

### The effect of p-JNK on α-SNC secretion does not require the transcriptional activity of cJUN

**Fig. 4:**
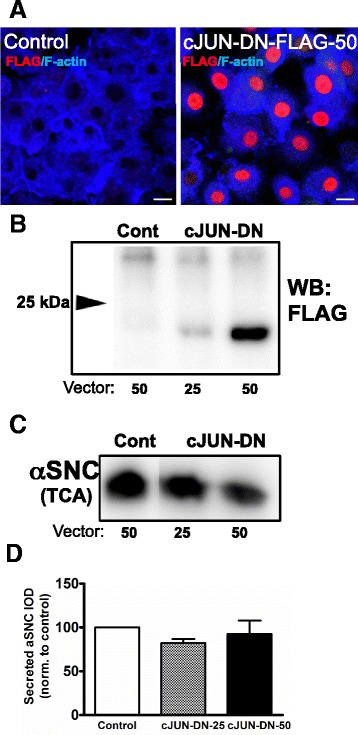
p-JNK effects on α-SNC secretion do not depend on the transcriptional activity of cJUN. PC12 cells expressing α-SNC/p25α were differentiated with NGF and transduced with control vector or a dominant-negative cJUN-Δ169-FLAG construct in doses of viral supernatant of 25 or 50 μl before transgene (α-SNC/p25α) induction with doxycycline. **a** Four days post-infection cells were prepared for immunofluorescence with anti-FLAG antibodies as indicated. Alexa647-conjugated phalloidin (F-actin) was used as counter stain. *Bar*, 10 μm. **b** Cell lysates were prepared for western blotting with anti-FLAG mAb for the detection of cJUN-Δ169-FLAG. *Arrow* indicates 25 kDa weight marker. The WB is the representative of two independent experiments. **c** Conditioned medium from transduced cells as above was analyzed for α-SNC by western blotting. All lanes were derived from the same membrane. **d** Normalized mean and SEM integrated optical density of α-SNC western bands above for four independent experiments

cJUN is an important transcription factor downstream of JNK activation, and as shown in Fig. 1a, the persistent JNK activation correlated with the activation of cJUN by phosphorylation. We therefore asked whether cJUN-induced transcription is important for the effects of p-JNK on α-SNC secretion. For this purpose, PC12 cells expressing α-SNC/p25α were transduced with a FLAG-tagged and truncated dominant-negative cJUN-∆169 construct, which retains DNA binding, but has lost its transactivation domain. Figure 4a, b shows that cJUN-∆169-FLAG was efficiently transduced into the vast majority of cells and expressed as a protein of the expected size (ca. 20 kDa), which localized to the nucleus. However, we found that dominant-negative cJUN did not significantly alter the level of secreted α-SNC to the medium (Fig. 4c, d), and we conclude that the effect of p-JNK on α-SNC release is not related to cJUN-induced transcriptional effects.

### Differentiated PC12 neurons expressing α-SNC activate microglia cells in co-culture to produce oxidants

**Fig. 5:**
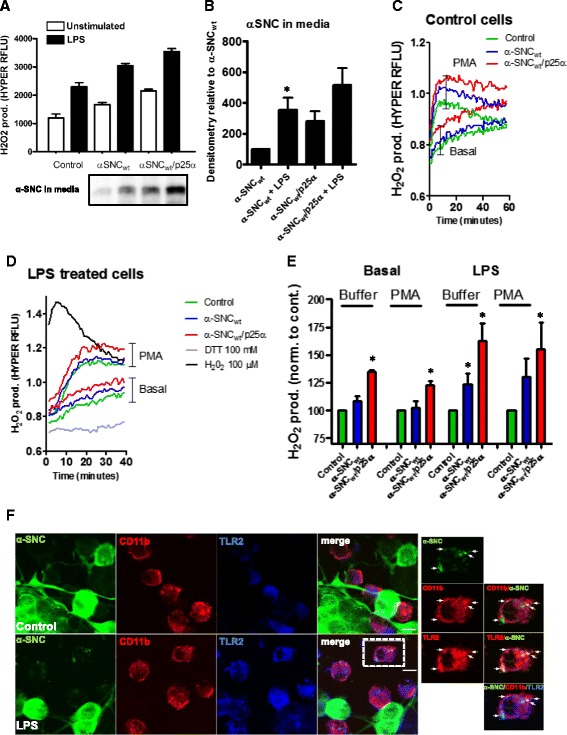
PC12 cells expressing α-SNC activate microglia cells to produce oxidants. **a** PC12 cells expressing α-SNC or α-SNC/p25α were co-cultured with unstimulated or LPS (100 ng/ml)-primed Ra2 microglia expressing the H_2_O_2_ biosensor HYPER3 overnight prior to the measurement of HYPER3 fluorescence by flow cytometry. Data are presented as mean relative fluorescent units (RFLU) +SEM. Conditioned medium was collected for WB analyses of α-SNC (*inset*). Data and representative image from three independent experiments. **b** Quantification of α-SNC in conditioned media from **a**. Data are presented as mean + SEM normalized to α-SNC-expressing PC12 cells (α-SNC) (*p < 0.05, *N* = 3). **c** Conditioned media were collected from PC12 cells expressing α-SNC or α-SNC/p25α after 2 days on transgene expression and transferred to Ra2 microglia expressing HYPER3. After a 2-h incubation, HYPER3 fluorescence was measured for 60 min without (Basal) or with 100 ng/ml PMA to activate NADPH oxidase. Data from a single experiment performed in triplicate wells is presented as a 485/420 nm ratio of Hyper3 fluorescence (RFLU). **d** Same as **c** but microglia were exposed to LPS (100 ng/ml) overnight prior to challenge with PC12-conditioned medium. 100 mM DTT or 100 μM H_2_O_2_ was added to obtain emission of the fully reduced/oxidized HYPER3 biosensor probe for comparison. Data from a single experiment performed in triplicate wells is presented as a 485/420 nm ratio of HYPER3 fluorescence (RFLU). **e** Bar graphs showing quantification of maximal oxidant response from (**c**–**d**). Data are presented as mean + SEM percent normalized to control cells (*p < 0.05, *N* = 3). **f** PC12 neurons expressing α-SNC were co-cultured with Ra2 microglia in the absence (*top row*, control) or presence of 100 ng/ml LPS (6 h, *bottom row*). Immunofluorescence stain shows the distribution of α-SNC, CD11b, and TLR2 as indicated. *Right side panels* show outlined cell in presence of LPS at higher magnification to demonstrate colocalization of α-SNC with CD11b and in particular TLR2. *Bar*, 10 μm

It has recently been shown that oligomeric α-SNC via binding to TLR2 or CR3 can activate microglia to produce inflammatory cytokines (e.g., TNFα and interleukin-1β) and mediate chemotaxis [40, 41]. The phagocyte NADPH oxidase (NOX2) is activated in response to many different stimuli, and the degree of either activation (superoxide production) or priming (hyper-responsiveness to stimuli) is a very sensitive measure of cell activation. When we incubated Ra2 microglia expressing the genetically encoded, fluorescent H_2_O_2_-sensor HYPER3 [45] with differentiated PC12 cells expressing either α-SNC_wt_ or α-SNC_wt_/p25α in direct co-culture, we found that PC12 nerve cells expressing α-SNC, and even more so α-SNC/p25α, enhanced microglial NADPH oxidase activity compared to microglia monoculture. When microglia were primed with LPS (100 ng/ml), HYPER3 fluorescence was augmented further and again showed a p25α-dependent increase. Analysis of the conditioned media from these co-cultures showed that regardless of p25α expression, secretion of α-SNC was upregulated by LPS stimulation of the microglia (*p* < 0.05, Fig. 5a, b). The factor(s) emitted from neurons to activate microglia were soluble mediators as the effect could be replicated by passive transfer of PC12 conditioned medium to the Ra2 microglia (Fig. 5c–e). Resting or LPS-stimulated Ra2 microglia expressing HYPER3 were incubated with conditioned medium from PC12 cells expressing α-SNC with or without p25α for 2 hours before basal or PMA-induced oxidant production was measured by ratiometric HYPER3 fluorescence in a microtiter plate reader. Again, we found that PC12 cells expressing α-SNC increased microglial oxidant production, and this was further increased by p25α co-expression either under basal or PMA-stimulated conditions (Fig. 5c–e). Microglia cells incubated with LPS were particularly susceptible to activation by conditioned medium from α-SNC-expressing PC12 cells compared to control cells (*p* < 0.05) (Fig. 5e). Thus, substance(s) released from synucleinopathic differentiated neurons can activate microglia. It has been described that both CD11b [41] and TLR2 [40] function as signaling and/or phagocytic receptors of α-SNC. We therefore conducted co-culture experiments with or without LPS activation of microglia and then performed immunofluorescence to gauge internalization of α-SNC and potential interaction with these surface receptors. As shown in Fig. 5, the increased secretion of α-SNC afforded by LPS activation of microglia correlated with uptake into Ra2 microglia, where colocalization with TLR2 was evident (arrows in Fig. 5f).

### Activated microglia release substance(s) that activate neuronal JNK signaling and increase neuronal α-SNC secretion

**Fig. 6:**
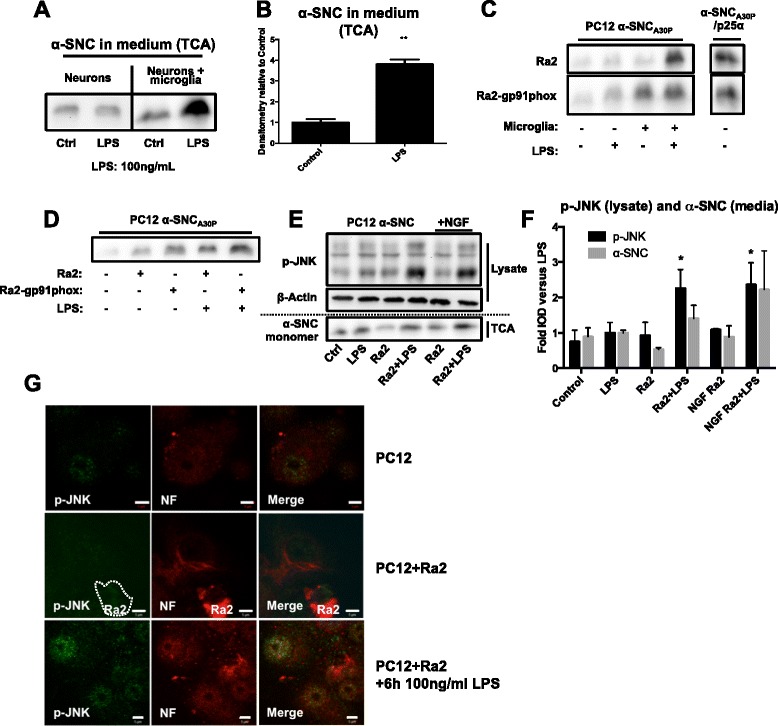
LPS-activated microglia increase neuronal α-SNC secretion and JNK phosphorylation. **a** PC12 cells expressing α-SNC were incubated in monoculture (neurons) or together with primary microglia isolated from neonatal rats (neurons + microglia) with or without 100 ng/ml LPS overnight. Conditioned medium was then analyzed for secreted α-SNC. The blot shown is representative of four independent experiments. **b** Quantification of **a** secreted α-SNC mean fold increase + SEM relative to control (**p < 0.01 compared to control, *N* = 4). **c** PC12 cells expressing α-SNC_A30P_ or α-SNC_A30P_/p25α were cultured together with Ra2 microglia or Ra2 overexpressing gp91^phox^ (Ra2-gp91^phox^) for increased oxidant production. Conditioned media was analyzed for secreted α-SNC. Images are representative of two independent experiments. **d** Media collected from Ra2 or Ra2-gp91^phox^ microglia with or without overnight exposure to 100 ng/ml LPS were transferred to differentiated PC12 cells expressing α-SNC_A30P_ in monoculture. After overnight incubation, the conditioned medium was collected and analyzed by western blotting. The blot shown is representative of two independent experiments. **e** Ra2 microglia were exposed to 500 ng/ml LPS in HBSS (Ra2 + LPS) with or without NGF for 6 h before transferring Ra2-conditioned HBSS to differentiated PC12 cells expressing α-SNC in monoculture. After 6 h of incubation, whole cell lysates and conditioned media were collected for WB analysis of p-JNK and secreted α-SNC, respectively. The blot shown is representative of three independent experiments. **f** Quantification of p-JNK (lysate) and α-SNC (TCA) in **e**. Mean fold increase + SEM relative to LPS alone (*p < 0.05 compared to “Ra2”; ^#^ *p* < 0.05 compared to “NGF Ra2”). **g** PC12 cells expressing α-SNC_wt_ in monoculture (PC12) or co-culture with Ra2 microglia (PC12 + Ra2) were incubated with 100 ng/ml LPS for 6 h prior to fixation and analysis for p-JNK and neurofilament (NF) by immunofluorescence. Slides were examined in a LSM510 confocal laser microscope. Representative images from two to three independent experiments are shown. *Bars*, 5 μm

As seen in Fig. 5a, b, LPS activation of microglia in co-culture greatly increased the secretion of α-SNC from neurons regardless of p25α expression. We further validated these results with primary rat microglia. Figure 6a, b shows that primary microglia activated by LPS caused a fourfold increased release of α-SNC from PC12 cells expressing α-SNC alone (*p* < 0.05 compared to control), again without excessive cell death as measured by LDH release (data not shown). The microglia redox state is intimately tied to microglia activation [52]. Therefore, we also analyzed the effect of overexpression of gp91^phox^, the rate-limiting subunit of the cyt b_558_ flavocytochrome complex of NADPH oxidase, in Ra2 microglia. As seen in Fig. 6c, gp91^phox^ overexpression in microglia caused an increased secretion of α-SNC from neurons in direct co-culture both in basal or LPS-primed states of the microglia. The effect of gp91^phox^ in microglia on neuronal secretion of α-SNC was not due to a direct effect of (short-lived) oxidants on the nerve cells, as the effect could be replicated by passive transfer of microglia conditioned medium to neuron monoculture (Fig. 6d). Secondly, we addressed if microglial signaling to increase neuronal α-SNC secretion was associated with neuronal JNK activation. To assess p-JNK levels accurately, these assays were performed in the absence of serum to avoid JNK activation associated with mitogenic signaling. Differentiated PC12 cells expressing α-SNC in monoculture were exposed to conditioned media from Ra2 microglia monocultures +/−LPS (100 ng/ml) exposure (Fig. 6e, f) with or without the addition of NGF. NGF has been shown to be important for maintaining PC12 cells in a differentiated state and to prevent apoptosis [53]. In agreement with previous experiments (Fig. 6a, b), we found that LPS alone had either no or little effect on neuronal JNK phosphorylation and α-SNC secretion. When differentiated PC12 cells were exposed to conditioned medium from control Ra2 microglia, there was no change in p-JNK levels but a decrease (although not statistically significant) in α-SNC secretion. However, conditioned medium transferred from inflammatory Ra2 microglia activated with LPS caused a statistically significant increase in p-JNK levels (*p* < 0.05 compared to PC12 cells with unexposed Ra2) associated with an increased α-SNC release, which in serum-free conditions is somewhat lower than in the presence of serum (compare Figs. 5b with 6f). The presence of NGF did not alter the response (Fig. 6f). Lastly, we performed confocal imaging with differentiated PC12 cells expressing α-SNC_wt_ and Ra2 microglia +/−LPS in co-culture to verify by independent methods that LPS-stimulated Ra2 microglia indeed caused an increase in p-JNK immunoreactivity in PC12 nerve cells (Fig. 6g). LDH release to medium was measured to estimate cell death (data not shown). In all cases, LPS exposure or microglia conditioned medium did not cause a biologically significant increase in cell death (the highest measure was a mean increase of Δ3.3 ± 0.9 % (*p* < 0.05) for cells exposed overnight to conditioned medium from LPS-primed microglia compared to control cells in monoculture).

### TNFα exposure increases neuronal JNK phosphorylation and α-SNC secretion

**Fig. 7:**
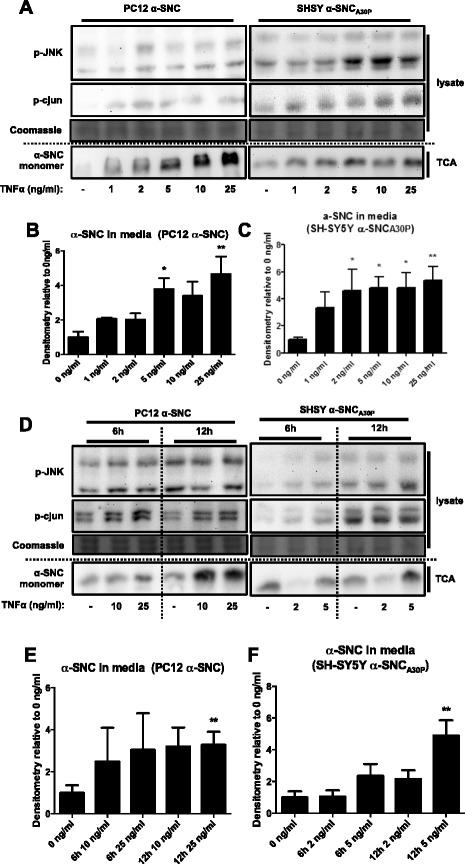
TNFα stimulation suffices to increase neuronal JNK phosphorylation and α-SNC secretion. **a** PC12 and SH-SY5Y cells expressing α-SNC and α-SNC_A30P_, respectively, were stimulated with TNFα at indicated concentrations for 18 h. Whole cell lysate and conditioned media (TCA) were collected and analyzed by WB. The blot shown is representative of three independent experiments. **b** Quantification of α-SNC in conditioned media from PC12 cells in **a** (*p < 0.05 and **p < 0.01 compared to “0 ng/ml”). Data are presented as mean fold increase + SEM (*N* = 3) relative to “0 ng/ml” control. **c** Quantification of α-SNC present in media from SH-SY5Y cells in **a** (*p < 0.05 and **p < 0.01 compared to “0 ng/ml”). Data are presented as mean fold increase + SEM (*N* = 3) relative to “0 ng/ml” control. **d** PC12 and SH-SY5Y cells expressing α-SNC and α-SNC_A30P_, respectively, were stimulated with TNFα at indicated concentrations for 6 and 12 h. Whole cell lysate and conditioned media (TCA) were collected and analyzed by WB. The blot shown is representative of three independent experiments. **e** Quantification of α-SNC present in conditioned medium from PC12 cells in **d** (**p < 0.01 compared to “0 ng/ml”). Data are presented as mean fold increase + SEM (*N* = 3) relative to “0 ng/ml” control. **f** Quantification of α-SNC present in media from SH-SY5Y cells in **d** (**p < 0.01 compared to “0 ng/ml”). Data are presented as mean fold increase + SEM (*N* = 3) relative to “0 ng/ml” control

The pro-inflammatory cytokine TNFα is an important effector of inflammatory microglia and is associated with disease progression in animal models of PD [39]. Therefore, we wanted to investigate if exogenously added TNFα could influence neuronal p-JNK levels and α-SNC secretion. Differentiated PC12 or SH-SY5Y cells were exposed to recombinant rat or human TNFα, respectively, in a concentration (Fig. 7a–c)- and time (Fig. 7d–f)-dependent manner. In differentiated PC12 and SH-SY5Y cells expressing α-SNC_wt_ or α-SNC_A30P_, respectively, there was a TNFα dose-dependent increase in α-SNC secretion to the medium accompanied by an increase in p-JNK and its downstream target p-cJUN levels after 18 h exposure (Fig. 7a–c). In both cell types, exposure to TNFα for 18 h in the low ng/ml range (2–5 ng/ml) led to a significant increase in α-SNC secretion compared to control (0 ng/ml TNFα) (*p* < 0.05). Two concentrations of TNFα were chosen for 6 and 12 h exposures of differentiated PC12 cells expressing α-SNC_wt_ (10 and 25 ng/ml) and differentiated SH-SY5Y cells expressing α-SNC_A30P_ (2 and 5 ng/ml) (Fig. 7d–f). Already after 6-h exposure to the highest TNFα concentration, we detected a slightly increased α-SNC level in the conditioned media of both PC12 and SH-SY5Y cells. After 12 h of exposure to the highest concentration, there was a clear and statistically significant increase in α-SNC levels in the media of both differentiated PC12 and SH-SY5Y cells (*p* < 0.05) (Fig. 7e–f). The increased levels of secreted α-SNC were correlated with increased p-JNK levels in the whole cell lysates (Fig. 7d). Additionally, when comparing conditions with low and high TNFα concentrations, it appeared that within each time point was a dose-dependency as observed above. Collectively, the data indicate that direct exposure of neurons to TNFα suffices to increase JNK activation and α-SNC secretion.

### JNK is required for TNFα to augment α-SNC secretion

**Fig. 8:**
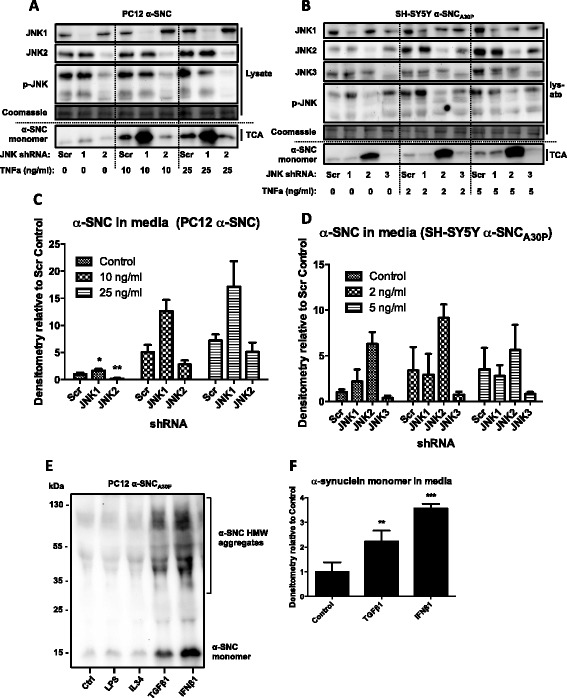
TNFα-induced α-SNC secretion from neurons requires JNK2 or JNK3, and non-inflammatory cytokines can increase α-SNC secretion. **a** PC12 cells expressing α-SNC and JNK1, JNK2, or scrambled control (Scr) shRNA were stimulated with TNFα (10 or 25 ng/ml) overnight. Whole cell lysate and conditioned media (TCA) were collected and analyzed by WB. Showing representative images from three independent experiments. **b** Differentiated SH-SY5Y cells expressing α-SNC_A30P_ and JNK1, JNK2, JNK3, or scrambled shRNA were stimulated with TNFα (2 or 5 ng/ml) overnight. Whole cell lysates and conditioned media (TCA) were collected and analyzed by WB. The blot shown is representative of three independent experiments. **c** Quantification of secreted α-SNC (TCA) in **a**. Data are presented as mean fold increase + SEM (*N* = 3) relative to control “Scr” shRNA (*p < 0.05 and **p < 0.01 compared to Scr shRNA in “Control.” *p* values are based on comparison with Scr shRNA within each group, i.e., 10 or 25 ng/ml). **d** Quantification of secreted α-SNC (TCA) in **b**. Data are presented as mean fold increase + SEM (*N* = 3) relative to control “Scr” shRNA (**p < 0.01 compared to “Control”). **e** PC12 cells expressing α-SNC_A30P_ were stimulated overnight with 100 ng/ml LPS or 10 ng/ml IL34, TGFβ1, or IFNβ1. Conditioned media (TCA) were collected and analyzed by WB. Showing representative image from three independent experiments. **f** Quantification of monomeric α-SNC in **e**. Data are presented as mean fold increase ± SEM (*N* = 3) relative to “Control” (**p < 0.01 and ***p < 0.001 compared to “Control”)

To address the possibility of a causal relationship for JNK in the TNFα-mediated increase in α-SNC secretion from neurons, we knocked each of the three JNK isoforms down with shRNAs. Then differentiated PC12 and SH-SY5Y cells expressing JNK shRNAs together with α-SNC_wt_ or α-SNC_A30P_, respectively, were subjected to TNFα exposure (Fig. 8a–d). For each cell line, we chose two TNFα concentrations and since JNK3 is not present in PC12 cells, we used only JNK1 or JNK2 shRNA in PC12 cells. In all cases, p-JNK levels and JNK knockdown were confirmed by p-JNK and JNK immunoblotting of whole cell lysates. Stable expression of JNK2 or JNK3 shRNA decreased α-SNC secretion from PC12 α-SNC_wt_ and SHSY5Y α-SNC_A30P_ cells (Fig. 8c, d, control) indicating that JNK is involved in α-synuclein metabolism also in the absence of p25α expression (see Fig. 3). Expectedly (Fig. 7), TNFα mediated an increased α-SNC secretion, which correlated with the activation of JNK (Fig. 8a–d, scrambled shRNA [Scr]). Importantly, the specific knockdown of JNK2 in PC12 cells or JNK3 in SHSY-5Y cells reduced the ability of TNFα to increase α-SNC secretion from the nerve cells indicating that JNK activation is an essential component of inflammatory signaling-induced α-SNC secretion. Cell death was assessed by LDH assay. Exposure to 10 and 25 ng/ml recombinant TNFα in the presence of JNK1 shRNA in PC12 cells (Fig. 8a, c) significantly increased cell death from 3.1 % ± 0.7 to 23.9 % ± 7.2 (*p* < 0.05) and 28.6 ± 11.7 (*p* < 0.05 compared to controls), respectively. Exposure of SH-SY5Y cells expressing JNK2 shRNA to 2 and 5 ng/ml recombinant TNFα increased (although not statistically significant) cell death from 8.2 % ± 3.5 to 28.9 % ± 7.2 and 28.6 % ± 11.7, respectively. The large increases in α-SNC levels in the conditioned medium from these conditions (only) are therefore not due to an active release mechanism.

### Non-inflammatory cytokines can also modulate α-SNC secretion from neurons

Since exposure to the pro-inflammatory cytokine TNFα increased neuronal secretion of α-SNC, we also examined the effect of several non-inflammatory cytokines on neuronal α-SNC secretion. Differentiated PC12 cells expressing α-SNC_A30P_ in monoculture were incubated with interleukin (IL)34, transforming growth factor (TGF)β1, or interferon (IFN)β1 at a concentration of 10 ng/ml. TGFβ1 is considered a master negative regulator of inflammation; IFNβ1 is a classical anti-viral and anti-bacterial cytokine, while IL34, which binds to colony-stimulating factor-1 receptors present on PC12 cells, was recently shown to increase microglia-afforded neuroprotection in an animal model of Alzheimer’s disease [54]*.* In addition, we also added LPS previously shown to have no effect in monoculture (Fig. 6a–d). As seen in Fig. 8e, f, both TGFβ1 and IFNβ1 affected neuronal α-SNC secretion as seen by a statistically significant increase in α-SNC levels in the conditioned media from PC12 monocultures (*p* < 0.01 and *p* < 0.001, respectively, compared to control). Neither LPS nor IL34 affected secretion.

### The correlation between JNK activation and α-SNC secretion relates to specific stress conditions

**Fig. 9:**
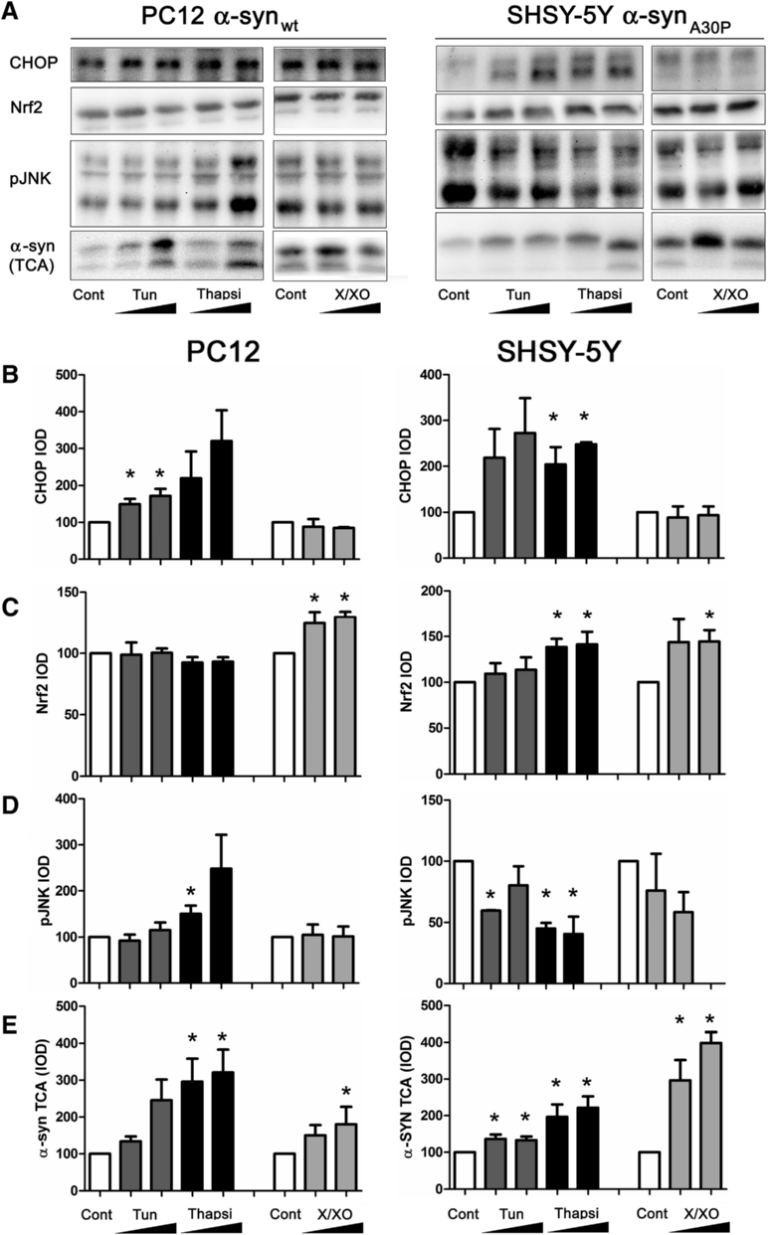
Lack of correlation between α-SNC secretion and JNK activation subsequent to ER stress or oxidative stress. **a** Differentiated PC12 cells expressing α-SNC or BDNF-differentiated SH-SY5Y cells expressing α-SNC_A30P_ were treated overnight with tunicamycin (250 and 500 ng/ml) or thapsigargin (50 and 75 nM for PC12 cells, 25 and 50 nM for SHSY-5Y cells) or xanthine/xanthine oxidase (50 μM/0.02 U/ml and 100 μM/0.02 U/ml) before collection of conditioned medium and cell lysates for western blotting as indicated. **b**–**e** Bar graphs show integrated optical density (IOD) of western blot bands from above as indicated for antigen and cell line. Results are expressed as mean normalized IOD relative to control (no treatment) ±SEM and represent data from three independent experiments (*p < 0.05 by Student’s *t* test compared to “Control”)

As a stress kinase and a mitogen-activated protein kinase, JNK is activated by many different factors and conditions in neurons. Therefore, we examined the universality of the correlation between activation of JNK and secretion of α-SNC into the surroundings. For the purpose, we imposed either ER stress with tunicamycin or thapsigargin exposure or alternatively oxidative stress generated by xanthine/xanthine oxidase production of superoxide, on PC12 and BDNF-differentiated SH-SY5Y cells expressing α-SNC_wt_ or α-SNC_A30P_, respectively. Both stress conditions are highly relevant in the context of neurodegenerative disease and PD. As shown in Fig. 9a–c, ER stress or oxidative stress specifically upregulated CHOP as a marker of ER stress (*p* < 0.05 tunicamycin in PC12 and thapsigargin in SH-SY5Y compared to Cont.) and Nrf2 as a marker of oxidative stress (*p* < 0.05 compared to control), respectively, in either cell type. The expression levels of transcription factor ATF3, which is transcriptionally regulated by Nrf2, closely followed the expression levels of Nrf2 in both cell types (data not shown). While ER stress induced with thapsigargin caused JNK activation in PC12 cells (*p* < 0.05 compared to control), both tunicamycin and thapsigargin moderately downregulated p-JNK in SH-SY5Y cells (*p* < 0.05 compared to control, Fig. 9a, d). Note that JNK in SH-SY5Y cells is activated differentially following ER-stress induction with thapsigargin depending on either ATRA (Fig. 2e, f) or BDNF differentiation (Fig. 9a, d)*.* In PC12 cells, both tunicamycin (not significant) and thapsigargin (*p* < 0.05 compared to control) increased α-SNC secretion to the medium, while preferentially thapsigargin caused α-SNC secretion in SH-SY5Y cells (*p* < 0.05). In neither cell type did oxidative stress under the experimental conditions cause activation of JNK, but nevertheless, α-SNC secretion was increased in both cell types particularly SHSY-5Y cells (*p* < 0.05, Fig. 9a, e). We therefore conclude that secretion of α-SNC under conditions of cellular stress (different from lysosomal perturbation and inflammatory signaling) does not necessarily correlate with JNK activation. Note that in the above experiment, released α-SNC may not necessarily derive from exophagy but could be due to other mechanisms of unconventional secretion. The essentiality of JNK2 and JNK3 for α-SNC secretion that we have described in the preceding sections therefore seems to be specifically tied to certain types of either intrinsic (autophagosomal and/or lysosomal perturbation) or extrinsic (inflammatory signaling) stress.

## Discussion

Within just a few years, a substantial body of evidence has emerged to support the idea that α-synucleinopathy can spread through the brain via release of proteotoxic α-SNC species from an affected neuron and uptake by a neighboring neuron [55]. Once internalized, these species can confer misfolding disease through proteopathic templating and thereby perpetuate the disease. The mechanism of neuronal α-SNC release is under intense investigation, and both late endosomes [11] and amphisomes [10], the organelles derived from fusion between autophagosomes and late endosomes, have been proposed as vesicular carriers of α-SNC to the surroundings by exocytosis. Note that exosomes, small intra-luminal vesicles contained within late endosomes and purported to contain or bind misfolded protein, are released in both cases. In the current study, we pinpoint active (phosphorylated) JNK in differentiated nerve cells as a pivotal regulator of α-SNC secretion by exophagy in response to both internal and external stress factors. With respect to the latter, we specifically show that classically activated microglia, and their inflammatory mediators such as TNFα, substantially augment α-SNC secretion from neurons.

### Active JNK is required for exophagy of α-SNC from differentiated neurons

We find that p25α, which partially inhibits fusion of late autophagosomal elements with lysosomes [10], caused persistent phosphorylation and activation of JNK accompanied by phosphorylation of downstream target cJUN to a much higher degree than α-SNC expression alone. However, pharmacological (SP600125) or genetic (JNK2/3 shRNA) JNK knockdown decreased, whereas constitutive active JNK signaling (MKK7B-JNK fusion constructs) increased α-SNC release from nerve cells regardless of p25α expression.

JNK1 has many “house-keeping” functions, while JNK2 and JNK3 are preferentially activated in response to cellular stress [33]. The mixed lineage kinase DLK is involved in the regulation of the pro-apoptotic versus physiological functions of the JNKs [34]. We show that knockdown of JNK2 in PC12 cells and JNK3 in SH-SY5Y cells significantly and robustly downregulated α-SNC release from cells co-expressing p25α (Fig. 3). Notably, JNK3 is not expressed in PC12 cells, and a certain degree of redundancy between JNK isoforms has been noted in global knockout studies in rodents [33] perhaps explaining the dependency on JNK2 in these cells. Conversely, of the constitutively active JNK1, JNK2, and JNK3 fusion constructs expressed in differentiated SH-SY5Y cells, JNK2 and JNK3 most effectively increased α-SNC secretion and, if any, JNK3 appeared to be the most efficient signaling JNK entity in relation to α-SNC release, as well as activation of endogenous JNK. Although JNK target cJUN was consistently phosphorylated in p25α-expressing cells, we find that its transcriptional activity is not required for the JNK effect on α-SNC secretion, as co-expression of dominant-negative cJUN did not alter α-SNC secretion (Fig. 4). This indicates that JNK2 and JNK3 modify the activity of cytosolic targets to effect increased exophagy.

Salubrinal inhibited both JNK activation and α-SNC secretion in PC12 nerve cells. However, expression of dominant-negative ASK1-K709R, which inhibits signaling through the classical ER-stress IRE1α-TRAF2-ASK1 signaling axis terminating in p38-mitogen-activated protein kinase and JNK activation [47], failed to decrease phosphorylation of JNK. For the same reasons, oxidative stress, which also signals through ASK1, is not the cause of JNK activation in p25α-expressing cells [50]. Instead, we consider that the mechanism of JNK activation following p25α-associated stress relates to the perturbation of lysosomal fusion with amphisomes [10]. Thus, in both PC12 and SH-SY5Y cells, bafilomycin, which more efficiently than p25α inhibits lysosomal fusion reactions, caused profound JNK activation (and α-SNC secretion). These observations are perhaps supported by drosophila studies showing JNK activation in response to accumulation of dysfunctional late endosomes [56]. The association of JNK isoforms with vesicular compartments through protein scaffolds or adaptor proteins that bind to either kinesin and dynein microtubule motors is well noted [57], and both cargo association [58–60] and mobility are regulated by JNK activity [59]. Thus, JNK3 in part determines directional organelle mobility in zebra fish axons [61] and has also been associated with aberrant directional cargo transport in response to pathogenic huntingtin as a basis of neurodegeneration [62]. The lack of retrograde movement of prelysosomal and lysosomal compartments may in turn directly favor exocytosis of these elements, because the fate of any endosomal, and presumably autophagosomal element, is to a large part determined by its distribution in the cell [63]. This agrees well with the observation that Rab8 overexpression, which dramatically increases α-SNC release from PC12 nerve cells [10], immobilizes LC3-positive autophagosomes/amphisomes at the cell periphery (unpublished data; FV).

Differences in subcellular distribution of JNK activity, isoform activation, and temporal activation patterns likely also explain the lack of strict correlation between JNK activation and α-SNC secretion under different stress conditions. For example, exogenous oxidative stress increases α-SNC secretion without any overt JNK activation in both cell lines, and in SH-SY5Y cells, ER stress imposed with tunicamycin and thapsigargin similarly increases α-SNC release under conditions where JNK activity is actually depressed. On the other hand, ER stress in PC12 cells increased both JNK activation and α-SNC secretion in a dose-dependent manner. However, we did not investigate the dependency of release on either JNK (isoforms) or autophagy in these alternative stress conditions. For now, we therefore specifically conclude that the increase in α-SNC secretion following either amphisome accumulation associated with lysosomal fusion deficiency or (select) cytokine signaling relies on the ability of JNK2 and JNK3 activation to promote exophagy of α-SNC.

### Reciprocal interactions between microglia and neurons activate microglia and increase α-SNC release from neurons

Microglia are essential for the development of full-blown neurodegenerative disease, but their contribution has mainly been relegated to the terminal phase of disease, where classically activated microglia exert neurotoxic effects by the production of reactive oxygen species [38] and other pro-inflammatory mediators [64]. However, in animal models as well as humans, microglia activation is widespread in the brain years or even decades before clinical symptoms. Differentiated PC12 nerve cells expressing α-SNC activated oxidant production of either resting or LPS-primed microglia in co-culture, as reported previously [65], and this was enhanced by the p25α co-expression in nerve cells, which increases α-SNC release. Microglial oxidant production was not only increased in direct co-culture, also passive transfer of neuron-conditioned media was able to induce increased microglial ROS production. While we have not sought to identify the neuronal factors responsible for microglia activation, it is reasonable to assume that the microglia are responding to released α-SNC oligomers and aggregates, which have recently been shown to engage TLR2 [40] and CR3 [41] scavenger receptors to activate microglia. In agreement herewith, we observe that internalized α-SNC in microglia colocalize in part with TLR2, and to a smaller degree with CD11b.

Activated microglia produce a range of inflammatory molecules that are neurotoxic at high concentrations. We show that classically activated (LPS; M1 activation), but not resting, microglia increase neuronal JNK phosphorylation and α-SNC secretion (in the absence of neuronal p25α expression). In co-culture, LPS-activated primary microglia from neonatal rats caused NGF-differentiated PC12 cells to secrete almost fourfold more α-SNC compared to controls. Passive transfer of conditioned media from microglial monocultures to neuronal monocultures allowed us to establish that the increased α-SNC secretion was accompanied by neuronal JNK phosphorylation. This experiment also excludes oxidants (because of their limited half-life) as the microglia-derived factor that directly influences neuronal p-JNK and α-SNC release. Thus, the potentiation of α-SNC secretion we observe in neurons exposed to conditioned medium from Ra2 microglia overexpressing gp91phox is likely a property of an altered microglial redox balance, which eventually supports inflammatory mediator expression [52]. One such classical factor secreted from activated microglia is TNFα [39, 52]. TNFα dose- and time-dependently significantly increased α-SNC secretion from both differentiated PC12 and SH-SY5Y cells by approximately two- to fourfold, and this correlated with JNK activation, again in the absence of p25α expression. Further, the increased α-SNC secretion afforded by TNFα required JNK, as shRNA knockdown of JNK2 in PC12 cells and JNK3 in SH-SY5Y cells reduced TNFα-induced α-SNC secretion. We note that JNK2 and JNK3 knockdown also under non-stimulated conditions (control cells) decrease α-SNC secretion in PC12 and SH-SY5Y cells, respectively, suggesting a basal mechanism. TNFα can be toxic to neurons at concentrations similar to the ones used here [66], but in our hands, there appeared no relevant toxicity (measured by LDH release and flow cytometric probes of cell death and membrane permeability), and morphological differentiation remained unaltered (or even slightly enhanced). The ability to induce an augmented release of α-SNC from PC12 nerve cells is not unique to TNFα or pro-inflammatory mediators: of a handful of cytokines tested with known neuronal receptors, anti/non-inflammatory cytokines TGFβ1 and IFNβ1, both increased α-SNC secretion from neurons in monoculture.

A few studies have documented that cytokines can induce autophagy [67], but we do not know how TNFα, or activated microglia for that matter, induce augmented neuronal α-SNC secretion by exocytosis of amphisomes. We do, however, implicate activated stress kinases JNK2 or JNK3 as essential components in the process. Of note, JNK activity is increased in post-mortem brains from PD patients [68]. Intriguingly, modest JNK inhibition in animal models of PD ameliorates motoric symptoms via unknown mechanisms [69, 70] that may relay to microglia activation [71] and/or conceivably to a mechanism as proposed above.

Rationally, any alterations in the exophagosomal release of α-SNC from neurons following exogenous stimulation would ultimately have to impinge on either autophagosomal uptake of α-SNC or on exocytosis of amphisomes. Future work is directed at understanding how JNK and cell non-autonomous mechanisms determine the burden of α-SNC released into the surroundings.

## Conclusions

In conclusion, we implicate stress kinases of the JNK family in the regulation of exophagy and release of α-SNC from nerve cells following both endogenous (p25α or bafilomycin-mediated disruption of autophagosomal flow) or exogenous stress (pro-inflammatory microglia and their cytokine products including TNFα). In a larger scope, our results insinuate that inflammatory microglia may harbor a so far unforeseen wickedness: in addition to inflicting direct bystander damage to neurons in late phases of inflammatory brain disease, they may also be essential mediators of early disease propagation by promoting the spread of proteopathic seeds.

## Abbreviations

ASK1: apoptosis signal-regulating kinase 1
CD: cluster of differentiation
CHOP: C/EBP homologues protein
CR: complement receptor
DLK: dual leucine zipper kinase
ER: endoplasmic reticulum
IFN: interferon
IL: interleukin
IOD: integrated optic density
IRE1α: inositol requiring enzyme-1α
JNK: cJUN-N-terminal kinase
LC3: microtubule-associated protein 1A/1B-light chain 3
LDH: lactate dehydrogenase
LPS: lipopolysaccharide
MKK7: mitogen-activated protein kinase kinase 7
NAPDH: nicotinamide adenine dinucleotide phosphate
NGF: nerve growth factor
PD: Parkinson’s disease
α-SNC: α-synuclein
TCA: trichloroacetic acid
TGF: transforming growth factor
TLR: Toll-like receptor
TNF: tumor necrosis factor
TRAF2: TNFa receptor-associated factor 2
UPR: unfolded protein response
WB: western blotting
